# A key gene, *violaxanthin de-epoxidase-like 1*, enhances fucoxanthin accumulation in *Phaeodactylum tricornutum*

**DOI:** 10.1186/s13068-024-02496-3

**Published:** 2024-04-02

**Authors:** Chenjie Li, Yufang Pan, Wenxiu Yin, Jin Liu, Hanhua Hu

**Affiliations:** 1 Key Laboratory of Algal Biology, Institute of Hydrobiology, Chinese Academy of Sciences, Wuhan, 430072 China; 2 University of Chinese Academy of Sciences, Beijing, 100049 China; 3 Key Laboratory of Poyang Lake Environment and Resource Utilization, Ministry of Education, and Center for Algae Innovation & Engineering Research, School of Resources and Environment, Nanchang University, Nanchang, 330031 China

**Keywords:** Diadinoxanthin, Fucoxanthin, Metabolic engineering, *Phaeodactylum tricornutum*, Strain screening, Violaxanthin de-epoxidase-like 1

## Abstract

**Background:**

Fucoxanthin has been widely investigated owing to its beneficial biological properties, and the model diatom *Phaeodactylum tricornutum*, possessing fucoxanthin (Fux) chlorophyll proteins as light-harvesting systems, is considered to have the potential to become a commercial cell factory for the pigment production.

**Results:**

Here, we compared the pigment contents in 10 different *P. tricornutum* strains from the globe, and found that strain CCMP631 (Pt6) exhibited the highest Fux content but with a low biomass. Comparison of mRNA levels revealed that higher Fux content in Pt6 was related with the higher expression of gene *violaxanthin de-epoxidase-like (VDL) protein 1* (*VDL1*), which encodes the enzyme catalyzing the tautomerization of violaxanthin to neoxanthin in Fux biosynthesis pathway. Single nucleotide variants of *VDL1* gene and allele-specific expression in strains Pt1 (the whole genome sequenced strain CCMP632) and Pt6 were analyzed, and overexpressing of each of the 4 *VDL1* alleles, two from Pt1 and two from Pt6, in strain Pt1 leads to an increase in downstream product diadinoxanthin and channels the pigments towards Fux biosynthesis. All the 8 *VDL1* overexpression (OE) lines showed significant increases by 8.2 to 41.7% in Fux content without compromising growth, and *VDL1 Allele 2* OE lines even exhibited the higher cell density on day 8, with an increase by 24.2–28.7% in two *Pt1VDL1-allele 2* OE lines and 7.1–11.1% in two *Pt6VDL1-allele 2* OE lines, respectively.

**Conclusions:**

The results reveal VDL1, localized in the plastid stroma, plays a key role in Fux over-accumulation in *P. tricornutum*. Overexpressing *VDL1*, especially *allele 2*, improved both the Fux content and growth rate, which provides a new strategy for the manipulation of Fux production in the future.

**Supplementary Information:**

The online version contains supplementary material available at 10.1186/s13068-024-02496-3.

## Background

Diatoms account for approximately 40% of the annual marine biomass production [1, 2]. Diatoms possess fucoxanthin chlorophyll proteins (FCP) as light-harvesting systems, and fucoxanthin (Fux) allows the organisms to capture blue-green light under deep-sea environments, thus enabling diatoms to thrive in the modern oceans. In addition, Fux has been widely investigated owing to its beneficial biological properties such as antioxidant, anti-obesity, anti-diabetic, anticancer, and antimicrobial activities [3–8]. *Phaeodactylum tricornutum*, a model species of diatoms with the well-characterized genome and abundant gene editing tools, is considered to have potential to become a commercial cell factory for Fux production [9].

Although the biosynthesis pathway of Fux has not been fully elucidated yet, several enzymes involved in steps from violaxanthin to Fux have been identified in *P. tricornutum*. In the first step, violaxanthin de-epoxidase-like (VDL) protein 1 (VDL1) catalyzes the tautomerization of violaxanthin to neoxanthin in tobacco leaves and in vitro [10]. VDL2 catalyzes the conversion of diadinoxanthin (Ddx) to allenoxanthin, and zeaxanthin epoxidase 1 (ZEP1) performs the epoxidation of haptoxanthin to phaneroxanthin [11]. The final step of the pathway was catalyzed by carotenoid isomerase-like protein 5 (CRTISO5) [12]. In addition, several enzymes involved in the early pathway steps of carotenoid biosynthesis have also been identified in *P. tricornutum*. For example, only ZEP3 functions as a zeaxanthin epoxidase which catalyzes the conversion of zeaxanthin to violaxanthin, and ZEP2 most likely converts diatoxanthin (Dtx) to Ddx in the xanthophyll cycle of *P. tricornutum* [13]. Violaxanthin de-epoxidase (VDE) catalyzes the conversion of Ddx into Dtx [14]. Furthermore, roles of genes encoding 1-deoxy-D-xylulose 5-phosphate synthase (DXS), phytoene synthase (PSY), phytoene desaturase (PDS), ζ-carotene desaturase (ZDS), and lycopene β-cyclase (LCYB) in carotenoid/Fux accumulation have been revealed [15–18].

*P. tricornutum* is generally considered to have the limited ecological relevance, and 10 accessions belonging to four genotypes were isolated at several locations worldwide [19, 20]. These *P. tricornutum* strains showed different growth rates, cell morphs and sizes, triacylglycerol contents, and temperature adaptability [19, 21–23], and their non-photochemical quenching (NPQ) capacity was also different more or less [24]. NPQ is associated with the xanthophyll cycle which involved the de-epoxidation of Ddx to absorb excess excitation energy generated during photosynthesis and dissipate it as heat in diatoms [25]. We compared the pigment contents in the 10 *P. tricornutum* strains, and strain CCMP631 (Pt6) exhibited the highest Fux content but with a low biomass. To interpret the over-accumulation of Fux in Pt6, a comparison of the transcript levels of genes involved in the Fux biosynthesis were performed between strains Pt1 (the whole genome sequenced strain CCMP632) and Pt6, and *VDL1* was found to keep a higher expression level till the stationary growth phase. In this study, single nucleotide variants (SNVs) of *VDL1* gene and allele-specific expression (ASE) in Pt1 and Pt6 were analyzed, the subcellular localization of VDL1 was determined, and four *VDL1* alleles from Pt1 and Pt6 were over-expressed, respectively, in strain Pt1. We obtained an engineered strain that possessed a Fux content comparable with that of strain Pt6 and higher biomass, which has potential application in Fux production.

## Results

### Screening of the Fux-enriched *P. tricornutum* strain

**Fig. 1:**
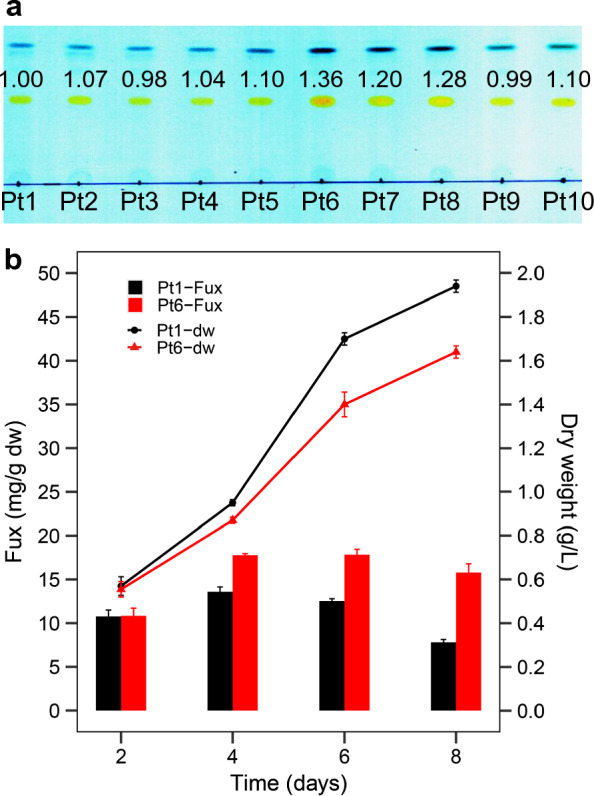
Fucoxanthin content in different accessions of *P. tricornutum*. **a** Comparison of fucoxanthin (Fux) content in *P. tricornutum* strains Pt1 ~ Pt10 by thin-layer chromatography. **b** Comparison of dry weight (dw) and Fux content in *P. tricornutum* strains CCMP2561 (Pt1) and CCMP631 (Pt6) cultivated in enriched nitrogen and phosphate medium

Since 1897, ten *P. tricornutum* ecotypes have been isolated from nine different geographic locations around the world with different habitats including sea shores, estuaries, rock pools, and tidal creeks [19]. We compared their growth and pigment content under the nitrogen-replete condition. Obviously, strains Pt6, Pt7 and Pt8, which belong to the same genetic clade [20], possessed relatively higher Fux content among the ten *P. tricornutum* strains (Additional file 1: Fig. S1). Strain Pt6, in particular, had the highest Fux content (Fig. 1a). After the initial screening, dry biomass and Fux content in Pt6 were further compared with those of strain Pt1, the whole genome sequenced strain [26]. The dry biomass on day 4, 6 and 8 was 9%, 21%, and 18% higher in Pt1, respectively, but the corresponding Fux content was 31%, 42% and 102% higher in Pt6 (Fig. 1b).

**Fig. 2:**
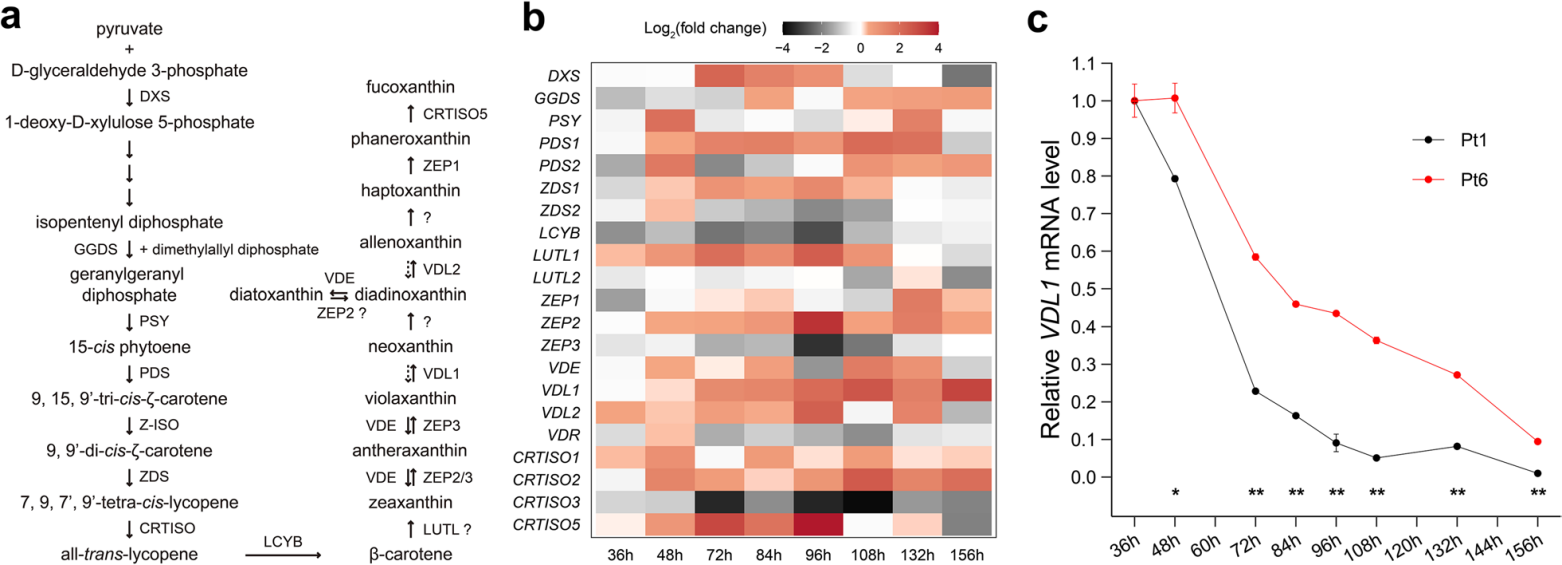
Transcript levels of genes encoding components involved in carotenoid biosynthesis in wild-type *P. tricornutum.* **a** Genes involved in fucoxanthin biosynthesis. **b** Transcriptional fold changes in strain CCMP631 (Pt6) relative to strain CCMP2561 (Pt1). **c** Relative *VDL1* mRNA levels in strains Pt1 and Pt6. Abbreviations: DXS, 1-deoxy-D-xylulose 5-phosphate synthase; PSY, phytoene synthase; GGDS, geranylgeranyl diphosphate synthase; PDS, phytoene desaturase; ZDS, ζ-carotene desaturase; LCYB, lycopene β-cyclase; LUTL, lutein deficient-like; ZEP, zeaxanthin epoxidase; VDE, violaxanthin de-epoxidase; VDL, violaxanthin de-epoxidase-like; VDR, VDE-related; CRTISO5, carotenoid isomerase 5. * and ** indicate statistically significant differences of *P* < 0.05 and *P* < 0.01, respectively

To reveal why Fux content was higher in Pt6, transcript levels of genes involved in carotenoid biosynthesis in wild-type *P. tricornutum* strains Pt1 and Pt6 were compared (Fig. 2a). Most of the genes showed higher mRNA levels in Pt6 throughout the whole growth phase (Fig. 2b), especially gene *VDL1*, whose mRNA level was 1.1 ~ 8.54-fold higher from 48 to 156 h (Fig. 2c).

### Sequence comparison of VDL1 in strains Pt1 and Pt6

Single nucleotide polymorphisms (SNPs) had been found in ten wild-type *P. tricornutum* strains Pt1–Pt10 [20], and allelic variation and allele-specific expression had also been observed in this diatom. According to the SNPs and INDELS of VDL1, the 10 strains were divided into four clades (Additional file 1: Fig. S2), consistent with the four genetic clades based on the genome resequencing data [20]. The coding region of Pt6 *VDL1* alleles (allele 1 and allele 2) boasted 11 SNVs (Additional file 1: Fig. S3), resulting in 6 amino acid differences between the encoded proteins (Additional file 1: Fig. S4). Pt1 *VDL1* allele 1 had an insertion of four nucleotides at position 138 before the translation initiation site, which might result in a shift of the translation initiation site, and the presence of additional 68 amino acids in its protein sequence (Additional file 1: Fig. S4) as annotated in JGI v2.0 (https://mycocosm.jgi.doe.gov/Phatr2) and Ensembl (https://protists.ensembl.org/Phaeodactylum_tricornutum). However, the sequence from -131 to -203 bp in the upstream of *VDL1* allele 1 was not transcribed according to the analysis of published RNA-seq data (Additional file 1: Fig. S5; Additional file 2: Table S1). In addition, 15 SNVs was detected in the coding region of Pt1 *VDL1* alleles 1 and 2 (Additional file 1: Fig. S3). Although the amino acid differences were found in the two alleles encoding VDL1 sequences of strains Pt1 and Pt6, predicted structures of the four VDL1 were very similar (Additional file 1: Fig. S6). To detect if the transcript of *VDL1* was affected by ASE, the published RNA-seq data of *P. tricornutum* strain Pt1 were analyzed. The results showed that transcript of *VDL1* increased gradually from 1 to 228 h during the whole cultivation period, but transcripts of alleles 1 and 2 showed no significant difference in response to nitrogen stress and culture time (Additional file 1: Fig. S7; Additional file 2: Table S2), and the average ratio of RPKM values of allele 1 versus those of allele 2 was more or less than 1. Seemingly, the higher Fux content in Pt6 might not be attributed to the variation of sequence and ASE in gene *VDL1*.

### Phylogeny and localization of *P. tricornutum* VDL1

**Fig. 3:**
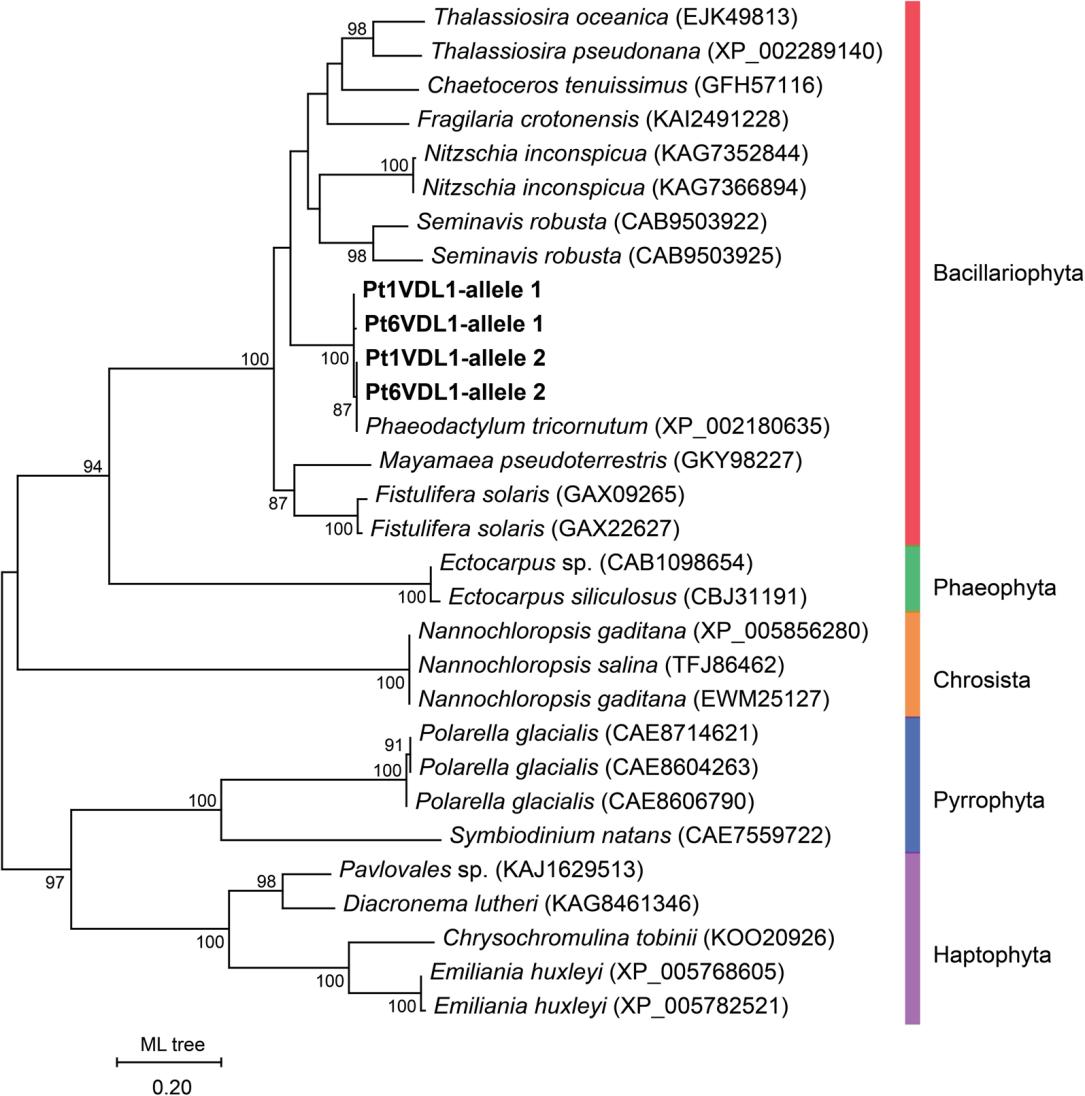
Phylogenetic relationships of VDL1 proteins from different species

To reveal the phylogenetic relationship of VDL proteins, 26 protein sequences were obtained by BLAST search against the NCBI protein database and a maximum likelihood tree was constructed. VDL1 sequences from the class Bacillariophyta (diatoms) formed a large clade, which was most closely related to the class Phaeophyta (brown algae). Diatom VDL1 was clearly distinct from those of classes Chromista (golden algae), Pyrrophyta (dinoflagellates), and Haptophyta (haptophytes) (Fig. 3). The similarity between *P. tricornutum* VDL1 and its homologue from other diatom species was above 60%, while the similarity of *P. tricornutum* VDL1 with those of species from the other four classes was below 50% (Additional file 2: Table S3).

**Fig. 4:**
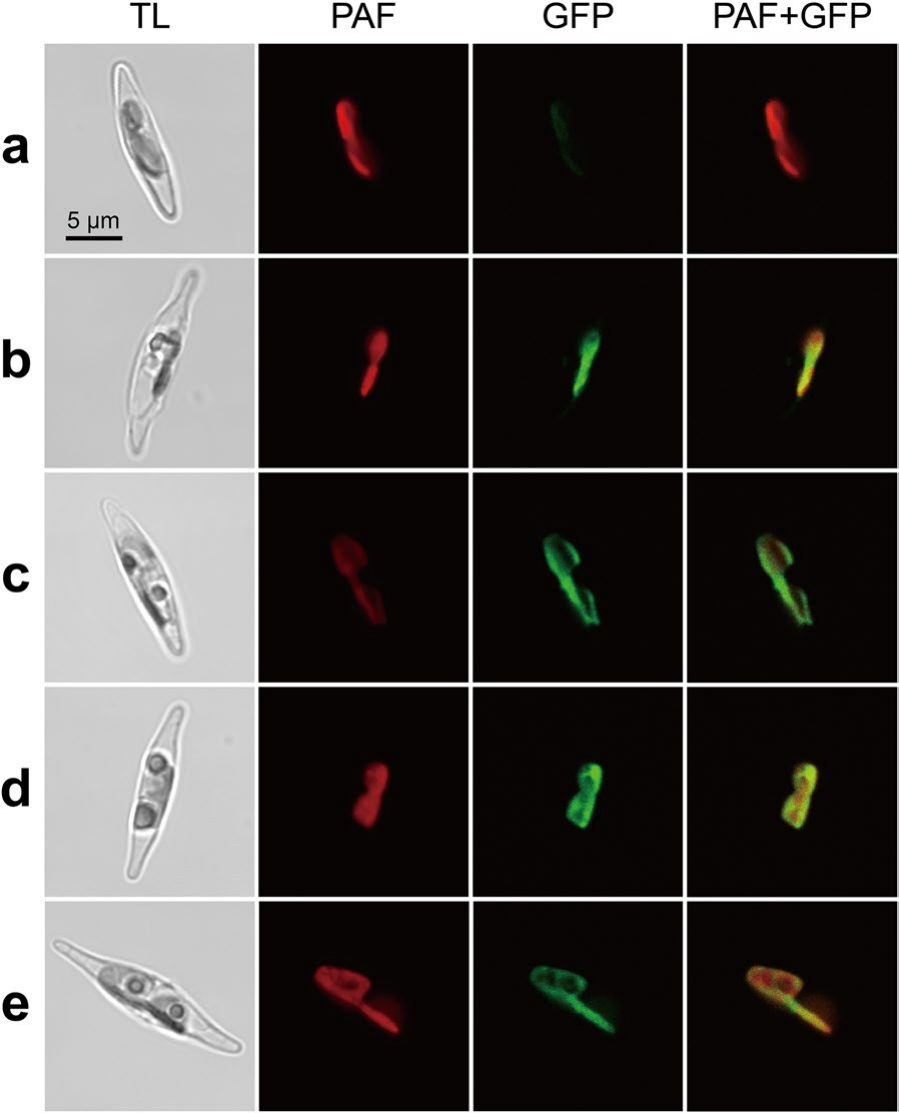
Fluorescent microscope images of cells expressing VDL1-eGFP. Panels show microscopical images of transmitted light (TL), chlorophyll autofluorescence (red, PAF), and GFP fluorescence (green), together with merged images (PAF + GFP) of chlorophyll and GFP fluorescence from left to right. **a** Wild-type Pt1. **b** Pt1VDL1-allele 1::eGFP. **c** Pt1VDL1-allele 2::eGFP. **d** Pt1VDL6-allele 1::eGFP. **e** Pt1VDL6-allele 2::eGFP

SignalP 6.0 and iPSORT predicted that Pt1 *VDL1* allele 1 encoded sequence contained no signal peptide or plastid targeting sequence. TargetP and HECTAR also predicted a low probability of a signal peptide in it (0.0011 and 0.1373, respectively), but Pt1 *VDL1* allele 2 and two Pt6 VDL1 alleles encoded sequences were predicted to have a signal peptide (Additional file 2: Table S4). However, when the four VDL1 alleles were fused with eGFP, respectively, and expressed in wild-type Pt1 cells, the fluorescence signal of eGFP (green) overlapped with the chlorophyll fluorescence (red) for all four fusion constructs, indicating the plastid localization of VDL1. In contrast, only chlorophyll fluorescence was observed in the wild-type control cells (Fig. 4).

### *VDL1* overexpression increased Fux accumulation without compromising growth

**Fig. 5:**
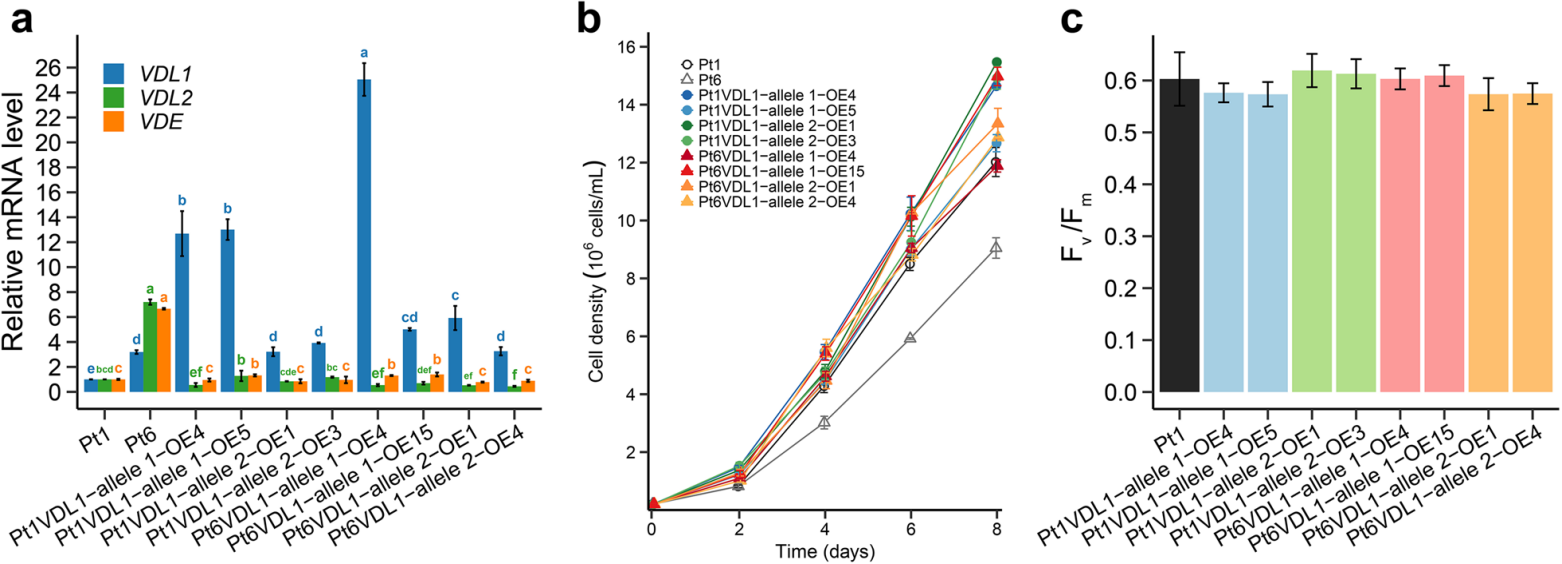
Relative mRNA levels of *VDL1*, *VDL2* and *VDE* detected by real-time quantitative PCR **a**, growth **b**, and photosynthetic efficiency (F_v_/F_m_) **c** of wild-type (Pt1 and/or Pt6) and *VDL1* overexpression (OE) strains. Allele 1 and allele 2 represent the *VDL1* alleles 1 and 2, respectively. Different letters above the bars indicate statistically significant differences (*P* < 0.05)

To overexpress *VDL1* in Pt1, four constructs containing sequence of *Pt1VDL1-allele 1*, *Pt1VDL1-allele 2*, *Pt6VDL1-allele 1*, and *Pt6VDL1-allele 2* were generated, respectively, and then were transferred into Pt1. Two overexpression (OE) lines were selected for each construct, and the relative mRNA expression levels of *VDL1* in all selected OE lines were significantly upregulated (3.2 ~ 25.0 fold) compared with that of Pt1, comparable to or even higher than that of Pt6 (Fig. 5a). However, the transcripts of *VDL2* and *VDE*, two genes with potential similarity in functions, changed hardly or even downregulated compared with wild-type Pt1 in OE lines (Fig. 5a). During the 8-day batch cultivation, the cell density of all OE lines was close to or higher than that of Pt1, especially on day 8, when Pt1VDL1-allele 2-OE1 had the highest cell density with a 28.7% increase relative to Pt1. In contrast, Pt6 had a 24.7% lower cell density than Pt1 on day 8 (Fig. 5b). There was no significant difference in the maximum photosynthetic efficiency between wild-type Pt1 and all OE lines (Fig. 5c).

**Fig. 6:**
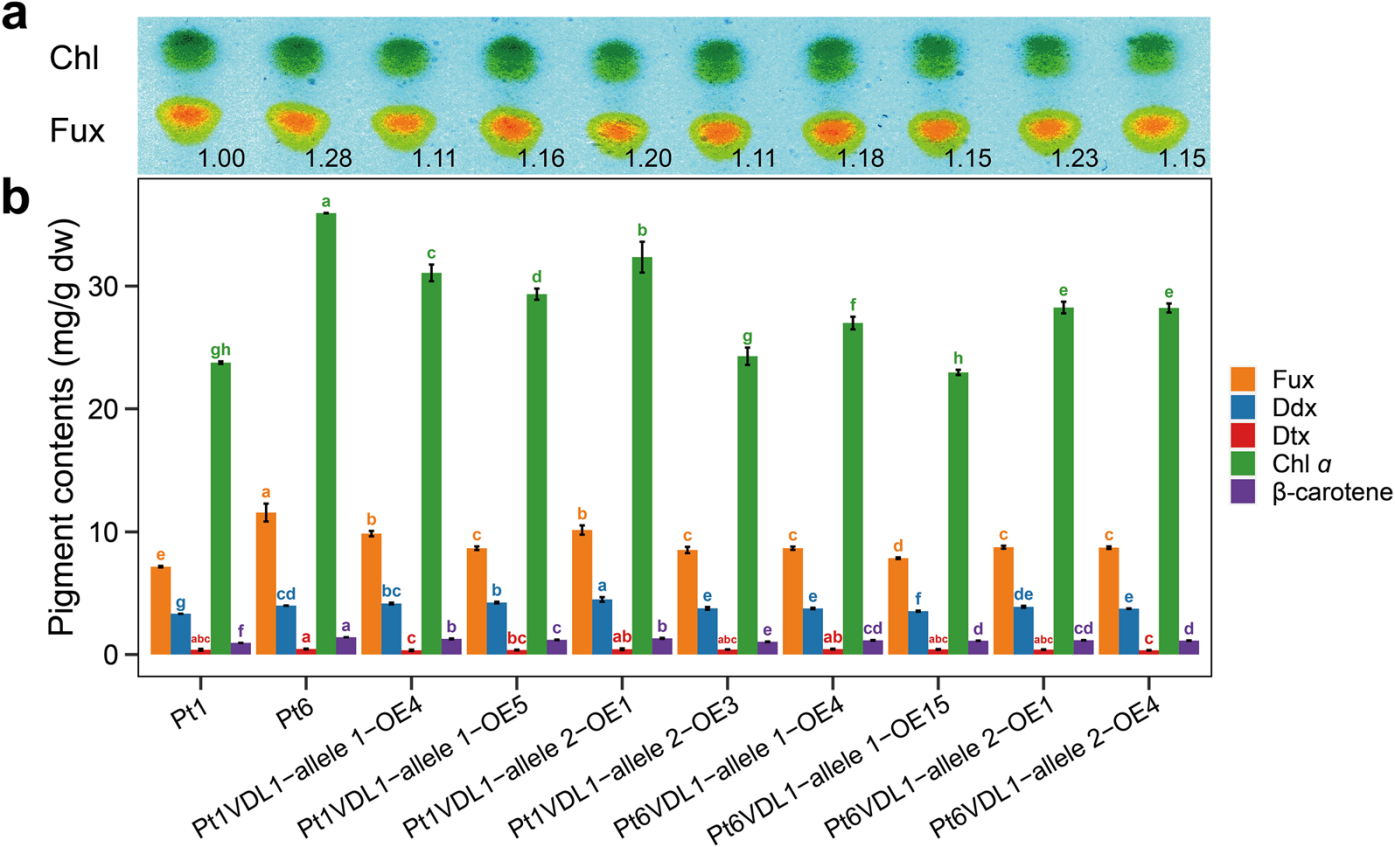
Pigment contents of wild-types Pt1 and Pt6, and eight *VDL1* overexpression (OE) strains. Allele 1 and allele 2 represent the *VDL1* alleles 1 and 2, respectively. **a** Thin-layer chromatography (TLC) analysis. The values above the TLC panel indicate the relative pigment content normalized to Pt1, which was set as 1. **b** HPLC analysis. Fux: fucoxanthin, Ddx: diadinoxanthin, Dtx: diatoxanthin, Chl a: chlorophyll a, β-carotene: β-carotene. Different letters above the bars indicate statistically significant differences (*P* < 0.05)

Under optimized culture conditions, Fux contents of all *VDL1* OE lines were higher than that of Pt1, but were slightly lower than that of Pt6 by thin-layer chromatography (TLC) analysis (Fig. 6a). High-performance liquid chromatography (HPLC) analysis showed that the relative content of Fux, β-carotene, and Ddx in all *VDL1* OE lines significantly increased by 8.2% to 41.7%, 6.6% to 32.6%, and 4.8% to 35.0%, respectively. In all OE lines, Pt1VDL1-allele 2-OE1 had the highest Fux content of 10.15 mg/g dry biomass, 42% higher than that of Pt1. All OE lines except two (Pt1VDL1-allele 2-OE3 and Pt6VDL1-allele 1-OE15) showed a significant increase of relative chlorophyll *a* content from 13.6% to 36.2%. However, strain Pt6 possessed the highest Fux content, reaching 11.57 mg/g dry biomass, which was 61.6% higher compared to Pt1 and 14% higher than that of Pt1VDL1-allele 2-OE1 line (an OE line with the highest Fux). Similarly, the highest relative content of chlorophyll *a* and β-carotene was also observed in Pt6 (Fig. 6b; Additional file 2: Table S5). Since the ratio of fucoxanthin to chlorophyll in *P. tricornutum* fucoxanthin chlorophyll protein (FCP) complex remains constant, it is reasonable that the chlorophyll content increases with the increasing fucoxanthin.

**Fig. 7:**
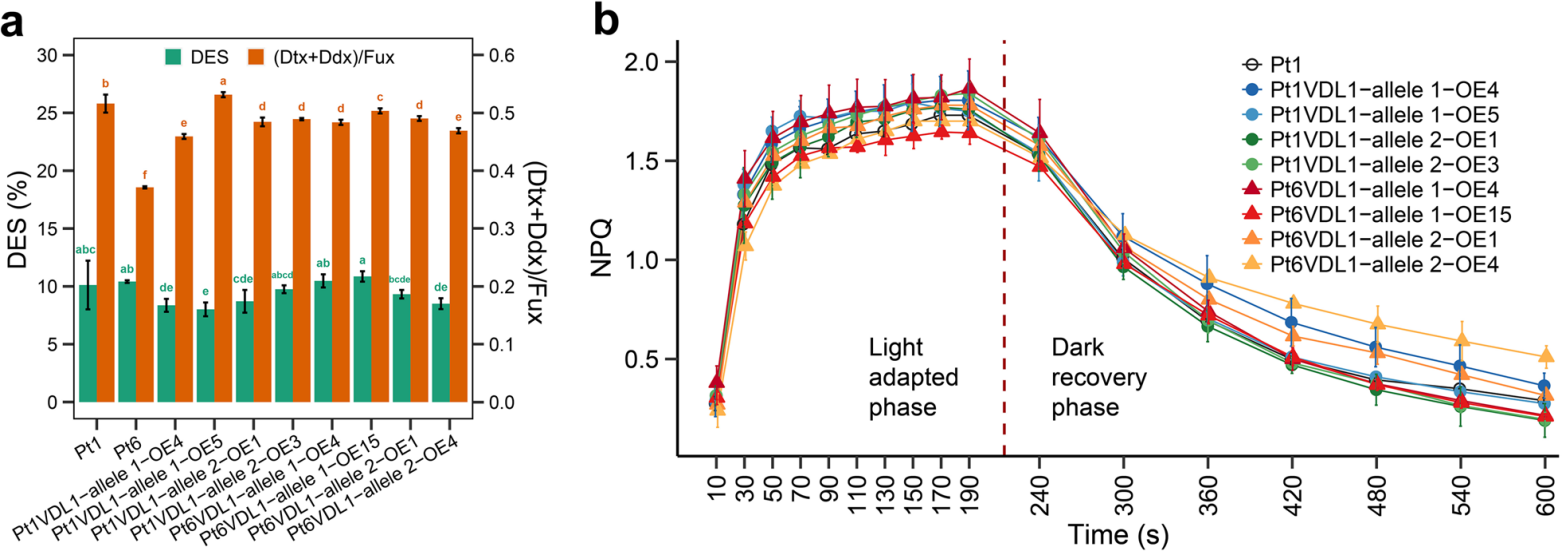
De-epoxidation state (DES) determined as Dtx/(Dtx + Ddx), relative level of the pool of xanthophyll (Dtx + Ddx) normalized to the fucoxanthin (Fux) content **a**, non-photochemical quenching (NPQ) kinetics **b** of wild-type (Pt1 and/or Pt6) and eight *VDL1* overexpression (OE) strains grown under low light (60 μmol photons m^−2^·s^−1^). Allele 1 and allele 2 represent the *VDL1* alleles 1 and 2, respectively. Ddx: diadinoxanthin, Dtx: diatoxanthin. Different letters above the bars indicate statistically significant differences (*P* < 0.01)

There were no significant differences in Dtx content between Pt1 and all OE lines, and the de-epoxidation state (DES) determined as Dtx/(Dtx + Ddx) was about 0.08 ~ 0.11, with significant (*P* < 0.01) decreases observed only in Pt1VDL1-allele1-OE5 line (Fig. 7a). In addition, relative level of the pool of xanthophyll (Dtx + Ddx) normalized to the Fux significant decreased in all OE lines except Pt1VDL1-allele 1-OE5 line. These results indicated that *VDL1* overexpression enhanced the synthesis of Ddx, the precursor of Fux and Dtx biosynthesis, and then Ddx flux mainly went to the Fux biosynthesis. Non-photochemical quenching kinetic profiles, mainly believed to be related with Dtx content, showed no significant changes in all *VDL1* OE strains and Pt1 (Fig. 7b) at low light, which also suggested *VDL1* overexpression had no effect on the synthesis of Dtx. However, the relaxation of NPQ got slow in strains Pt1VDL1-allele 1-OE4, Pt6VDL1-allele 2-OE4 and Pt6VDL1-allele 2-OE15, while the relaxation of NPQ in the other strains showed no significant difference to that of WT.

**Fig. 8:**
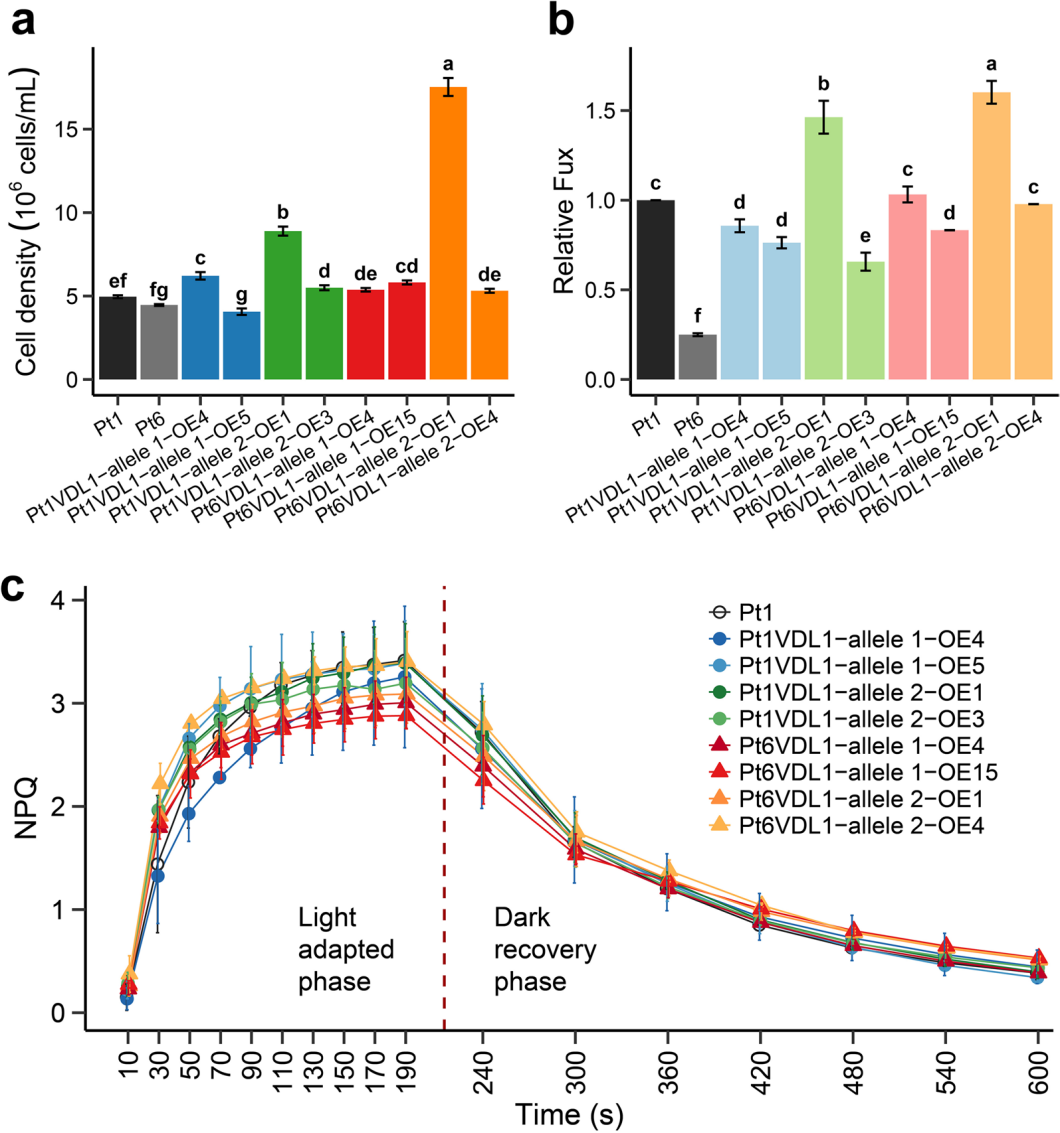
Cell density **a**, relative fucoxanthin (Fux) content at day 6 **b**, non-photochemical quenching (NPQ) kinetics **c** of wild-type (Pt1 and/or Pt6) and eight *VDL1* overexpression (OE) strains grown under high light (1000 μmol photons m^−2^·s^−1^). Allele 1 and allele 2 represent the *VDL1* alleles 1 and 2, respectively. Different letters above the bars indicate statistically significant differences (*P* < 0.05)

To further interpret the physiological roles of *VDL1* overexpression in *P. tricornutum*, growth, Fux content and NPQ at extremely high light was investigated. Under light irradiation conditions of 1000 μmol photons m^−2^ s^−1^, the occurrence of higher cell density (Fig. 8a) and fucoxanthin content (Fig. 8b) was only observed in transformants Pt1VDL1-allele 2-OE1 and Pt6VDL1-allele 2-OE1. However, the NPQ of the transformants did not show significant differences compared to the wild-type (Fig. 8c), though the NPQ of both the wild-type and transformants were significantly higher than that at low light (Fig. 7b). The results indicated that the increased fucoxanthin in *P. tricornutum* was favorable to the cell growth under high light condition, but a sole increase of fucoxanthin content would not affect NPQ.

## Discussion

According to the sequence analysis of *VDL1* allele genes from *P. tricornutum* accessions Pt1 and Pt6, an insertion of four bases was found in the sequence of *Pt1VDL1-allele 1* (Additional file 1: Fig. S3). Such an insertion was also observed in accessions Pt2, Pt3, Pt4, and Pt9 (Additional file 2: Table S6), suggesting a common origin of the mutation at this site. The phylogenetic tree constructed based on the *VDL1* gene polymorphism represented 10 *P. tricornutum* accessions arranged in four different clades, consistent with the clade classification based on whole-genome variations in Pt1-10. It seemed that *VDL1* was not a conserved gene and its mutation frequency was comparable to the overall mutation frequency of the species [20]. Distinct difference of Fux content was observed in Clades A to D, and Clade D (Pt6, Pt7, and Pt8) possessed the higher Fux content. Apparently, *VDL1* gene is the key gene for the regulation of Fux biosynthesis. Indeed, *VDL1* relative mRNA level was higher in Pt6 compared with Pt1, especially after exponential growth phase, which might contribute to the higher Fux content in Pt6. Most *cis*-regulatory elements located in the two *Pt6VDL1* alleles promoters were identical to those in the two *Pt1VDL1* alleles promoters (Additional file 2: Table S7), except an extra MYBHv1 binding site (CCAAT box) and an enhancer-like element involved in anoxic specific inducibility (GC-motif) present in *Pt6VDL1-allele 1* promoter (Additional file 1: Fig. S8). The differences might result in the higher expression level of *VDL1* in strain Pt6.

*P. tricornutum* VDL1 catalyzes the tautomerization of violaxanthin to neoxanthin in tobacco leaves and in vitro [10]. Neoxanthin is the precursor not only of the major light-harvesting carotenoid Fux but also of Ddx involved in photoprotection. The Ddx content in Pt6, a natural strain with higher *VDL1* mRNA level, was higher than that in Pt1. Overexpressing of each of the 4 *VDL1* alleles, two from Pt1 and two from Pt6, in strain Pt1 virtually leads to an increase in downstream product Ddx. However, there were no significant differences in the relative content of Dtx, which is directly related to photoprotection, among all OE strains. These results indicate that overexpression of *VDL1* only increases the total amount of pigments in the Ddx cycle pool, which is likely to enhance the photoprotection ability of the OE strains under strong light without affecting the transient NPQ capacity [27, 28]. Under unstressed conditions (F_v_/F_m_ ratio was close to 0.60), there was no significant difference in NPQ between the OE strains and Pt1, which further confirms that Dtx and DES were hardly affected by *VDL1* overexpression.

Although the relative content of Ddx + Dtx and that of Fux increased, the ratio of (Ddx + Dtx)/Fux decreased in 7 out of the 8 *VDL1* OE lines, which suggests that *VDL1* overexpression channels the pigments in the Ddx cycle towards Fux biosynthesis. It should be noted that an excellent characteristic to promote the total pool of pigments in the Ddx cycle qualifies *VDL1* OE strains as cell factories, for a stronger photoprotection ability may endow the lines with a greater advantage in Fux accumulation under high light intensity and prolonged illumination [29].

Proteomes revealed that carotenoid biosynthesis occurred in the plastid of *P. tricornutum*, and several key enzymes involved in Fux biosynthesis, including CRTISO5, ZEP1, ZEP2, ZEP3, VDE, and LUTL, were present in the purified plastid [30]. Although *P. tricornutum* VDL1 was not a predicted plastid protein by ASAFind, it was an assigned plastid protein by blasting against Arabidopsis proteome database in PPDB [30]. GFP fusion experiments in our study showed that VDL1 was localized in *P. tricornutum* plastid stroma. Therefore it is safe to conclude that the biosynthesis of Fux from β-carotene takes place in the plastid. Furthermore, targeting of PtVDL1 to the thylakoid lumen rather than plastid stroma led to higher activity of the enzyme in tobacco leaves [10].

Although Pt6 possesses higher Fux content, its growth rate and cell density were lower compared with Pt1 under standard f/2 nutrient condition. Overexpressing *VDL1* from Pt6 or Pt1 in Pt1 enhanced the Fux accumulation, but did not decrease the growth rate and cell density. In fact, on day 8 the cell density increased by 24.2–28.7% in two *Pt1VDL1-allele 2* OE lines, and 7.1–11.1% in two *Pt6VDL1-allele 2* OE lines, respectively. These results showed that elevated Fux would not affect the growth, and the low biomass in Pt6 should not be attributed to the higher *VDL1* transcript levels. In addition, most of the *VDL1* OE lines showed a significant increase in chlorophyll *a* and all OE lines showed an increase in β-carotene compared to wild-type Pt1. Chlorophyll *a* is involved in the formation of FCP and β-carotene is an important component of the photosystems. Increased relative content of FCP components including Fux, Ddx, and chlorophyll *a*, gave rise to the coordinated increase of other FCP components, thereby increasing the overall content of FCP without improving the photosynthetic efficiency (Fig. 5c). However, changes in photosystem components could ultimately stimulate the growth of the *VDL1* OE strains [29, 31].

After an extensive screening and comparison of different Pt strains from the globe, strain Pt6 was found to have the highest Fux content but slower growth, while Pt1 exhibited the opposite characteristics. Analysis of the transcript levels of genes involved in carotenoid biosynthesis pathway revealed that higher expression level of *VDL1* in Pt6 resulted in the higher Fux content. Overexpressing *VDL1* in Pt1 improved both Fux content and growth rate, and the genetically engineered strain has the potential cell factory to yield Fux.

## Methods

### Strains and growth conditions

Axenic cultures of *P. tricornutum* strains CCMP632 (Pt1) and CCMP631 (Pt6) were gifts from Chris Bowler and originated from the Provasoli-Guillard National Center for Marine Algae and Microbiota at Bigelow Laboratory for Ocean Sciences, USA. In batch static culture, *P. tricornutum* wild-types and the transformants were cultured in artificial seawater containing f/2 nutrient additions [32] with an initial cell density of 2 × 10^5^ cells/ml. The cultures were maintained under continuous light of 60 μmol photons/(m^2^·s) at 22 °C. Cultivation was performed in 250-ml Erlenmeyer flasks with 120 ml medium, and the static cultures were gently shaken every 6 h. The growth was monitored by microscopic cell counting. For Fux measurement, cultures were grown in the medium with the nitrate concentration of three times (2.64 mM), and the initial cell density was 6 × 10^5^ cells/ml in a 12 h/12 h light/dark illumination cycle.

For the high light experiments, cultivation was performed in a 50 ml ventilated cell culture flask containing 30 ml medium with 2.64 mM nitrate and then the flask was incubated in a shaker-incubator at 100 rpm with the initial cell density of 2 × 10^6^ cells/ml under a continuous light irradiation of 1000 μmol photons/(m^2^·s) at 20 °C (to ensure temperature around the flasks below 22 °C). NPQ was measured after 12 h exposure to the high light, and cell density and relative Fux content were determined on day 6.

### Sequences of *VDL1* and its polymorphism analysis among ten *P. tricornutum* strains

The raw genomic resequencing data of strains Pt1–10 were obtained through BioSample numbers SAMN08369620, SAMN08369621, SAMN08389622, SAMN0839623, SAMN0839624, SAMN01839625, SAMN08329626, SAMN03369627, SAMN08349628, and SAMN08339629 [20]. Low-quality reads were filtered using Fastp with default parameters [33]. The published genome assembly of *P. tricornutum* was used as the reference [26], and BWA was used to align the genomic resequencing data of the 10 *P. tricornutum* strains to the reference sequence [34]. The GATK tool was used to remove duplicate sequences and detect variants, including SNPs, small insertions and deletions [35]. Vcftools was used to filter the polymorphic sites [36], and the polymorphic sites of *VDL1* were selected. TASSEL was then used for sorting and format conversion of the *VDL1* polymorphic sites [37]. A maximum likelihood phylogenetic tree was constructed using FastTree with 500 bootstraps [38] based on the polymorphism of *VDL1*. The protein models of VDL1 were predicted by Alphafold2 and were viewed using PYMOL software. Information on *VDL1* polymorphic sites, and software running parameters and version is shown in Additional file 2: Table S6.

RNA-seq data of strain Pt1 with NCBI accession ID PRJNA38276 were used for the analysis of VDL1 ASE. Sequences of the two *VDL1* alleles (designated as *VDL1-allele 1* and *VDL1-allele 2*) were used as the reference sequence. Reads with remaining adapters or with a low quality value (Q ≤ 20 more than 30%) were discarded, and the clean data were aligned to the two *VDL1* alleles [39] and alignments without mismatch or gap were selected for subsequent statistical analysis. The number of reads matched to the two alleles was counted, respectively, and then RPKM values were calculated accordingly. In addition, the RNA-seq data were also used to calculate the coverage depth of the 203 bp before and the 101 bp after *VDL1* translation start site with Samtools version 1.9 [40].

*VDL1* gene and its upstream sequence (1180 bp) were amplified using primer pair *VDL1-Pst1-F* and *VDL1-Hind3-R* with Pt1 and Pt6 genomic DNA as templates. The PCR products were cloned into the pPhat-T1 [41] vector (between *Pst* I and *Hind* III) using the Clone Express Ultra one-step cloning kit (Vazyme, China), and then were sequenced with primers *pPhat1-P-VDL1-F* and *VDL1-326-R*. PlantCare (http://bioinformatics.psb.ugent.be/webtools/plantcare/html/) was used to analyze *cis*-acting regulatory elements [42] in the putative promoters (Additional file 2: Table S7) of *VDL1*.

### Subcellular targeting predictions and phylogenetic analyses

Subcellular targeting predictions ofVDL1 were carried out using SignalP 6.0 (https://services.healthtech.dtu.dk/services/SignalP-6.0/), iPSORT (https://ipsort.hgc.jp/), TargetP 2.0 (https://services.healthtech.dtu.dk/services/TargetP-2.0/), and HECTAR (http://www.sb-roscoff.fr/hectar/) [43–46]. Using the VDL1 protein sequence as a reference, similar protein sequences were retrieved via the NCBI online tool BLAST, and sequences with an *e*-value less than 10^–5^ were selected as the raw data for constructing the phylogenetic tree. The selected sequences were aligned using Mega11 (Version 11.0.13) and a phylogenetic tree was constructed based on the maximum likelihood method with 100 bootstrap replicates [47].

### Vector construction and transformation

To obtain the allele-specific overexpression (OE) vectors for *VDL1* gene, PCR was used to amplify the entire coding region of *VDL1* from cDNA of strains Pt1 and Pt6 using the primers *VDL1-KpnI-F* and *VDL1-XbaI-R*. The amplified fragments were then inserted into the plasmid pPhat-T1 [41] between the *Kpn* I and *Xba* I sites, resulting in *VDL1* OE vectors. The *VDL1* OE vectors were sequenced to screen the allele-specific OE vectors for strains Pt1 (Pt1VDL1-allele1-OE and Pt1VDL1-allele2-OE) and Pt6 (Pt6VDL1-allele1-OE and Pt6VDL1-allele1-OE). To analyze the subcellular localization of VDL1, we constructed the pPhaT1-VDL1-eGFP vector, which expressed a C-terminal eGFP fusion protein in *P. tricornutum* cells. The full-length sequences of *VDL1* from strains Pt1 and Pt6 were amplified using the primer pairs *VDL1-eGFP-long-EcoRI-F* and *VDL1-eGFP-XbaI-R*, and *VDL1-eGFP-short-EcoRI-F* and *VDL1-eGFP-XbaI-R*, respectively. The resulting PCR products were cloned into pPhaT1-linker-eGFP vector [48] between the *EcoR* I and *Xba* I sites and sequenced to obtain two allele-specific sequences of *VDL1* for strains Pt1and Pt6, respectively. The primers used for vector construction and corresponding maps are provided in Additional file 1: Fig. S9 and Additional file 2: Table S8.

Before electroporation into wild-type Pt1, the VDL1-OE vectors and VDL1-eGFP vectors were linearized using the *Nde* I and purified using a DNA purification kit, as previously described [49]. Transformants were screened based on the integration of *VDL1* gene. eGFP transformants at the exponential growth stage were observed using a Leica TCS SP8 laser scanning confocal microscope. The excitation wavelength for eGFP fluorescence and chlorophyll autofluorescence was 488 nm, with detection wavelengths of 500–550 nm and 650–690 nm, respectively [50].

### RNA extraction, cDNA synthesis and real-time quantitative PCR (RT-qPCR)

For detecting the relative expression levels of genes involved in the carotenoid biosynthesis, algal cells cultured for 4 days (OE lines) or at different growth phases were centrifuged at 3000 *g* for 10 min and then RNA was extracted using the Trizol reagent (TaKaRa, China). The first-strand cDNA was synthesized from RNA using the HiScript III First Strand cDNA Synthesis Kit (Vazyme, R310-01/02, China) and used as the template for RT-qPCR. RT-qPCR was performed with the LightCycler 480 SYBR Green I Master (Roche, Germany) and the LightCycler 480 Real-Time PCR System (Roche). The primers used for RT-qPCR are listed in Additional file 2: Table S8. *Histone H4* gene was used as an internal control gene [51], and the relative expression of the target gene was calculated according to the 2^−ΔΔCt^ method [52].

### Pigment extraction and analysis

Cell samples were collected by centrifugation at 3000 *g* for 10 min, and one aliquot of the sample was washed twice with a 0.5 M ammonium bicarbonate solution, dried in a vacuum drying oven at 80 °C for more than 24 h, and then weighed. Another aliquot of the sample was used for pigment extraction according to [53]. After washing twice with deionized water, fresh algal pellets were mixed with methanol containing 0.05% butylated hydroxytoluene (BHT) for 1 h, and subsequently vortexed with chloroform twice volume of the methanol for 1 h. 0.75% NaCl aqueous solution, one-quarter of the total volume, was added and then mixed. The chloroform layer was collected by centrifugation at 3000 *g* for 5 min. After drying the chloroform under nitrogen gas, the residue was redissolved with acetone. The acetone extracts were separated using a HPLC system equipped with a Waters 2695 separation module, a Waters 2996 photodiode array detector, and a Waters Spherisorb 5 µm ODS2 4.6 × 50 mm analytical column (Waters, Milford, MA, USA).

For TLC, 5 µL of the acetone extracts was loaded onto a pre-coated silica gel GF254 plate (Qingdao, China) with petroleum ether (boiling point 60–90 °C)/acetone (2:1, v/v) as the solvent system. After development for 20 min, the plate was air-dried and scanned, and the scanned images of the pigment spots were subjected to relative quantification analysis using ImageJ.

The relative Fux content was determined spectrophotometrically at 445, 663 and 750 nm using formula according to Wang et al. [54].

### Chlorophyll fluorescence measurement

The initial fluorescence emission level (F_0_) in the dark, maximum fluorescence emission level (F_m_) in the dark, and maximum fluorescence emission level (F_m’_) under illumination were determined using an Aqua Pen instrument (Photon Systems Instruments, Czech) at a wavelength of 455 nm. The maximum photosynthetic efficiency of PSII was calculated based on F0 and Fm using the formula (F_m_-F_0_)/F_m_ = F_v_/F_m_. Non-photochemical quenching (NPQ) was calculated as (F_m_-F_m’_)/F_m_ [55]. Prior to measurement, the samples were dark-adapted at 25 °C for 15 min.

### Supplementary Information

**Additional file 1: Figure S1.** Fucoxanthin content in different accessions of *P. tricornutum*. **Figure S2.** Phylogenetic association of *VDL1* gene in different accessions of P. tricornutum based on polymorphic sites (including SNP and INDELS) using a maximum likelihood approach. **Figure S3.** Single nucleotide variants (SNVs) of the *VDL1* gene in *P. tricornutum* strains CCMP2561 (Pt1) and CCMP631 (Pt6). **Figure S4.** Sequence alignment of the amino acids of VDL1 in *P. tricornutum* strains CCMP2561 (Pt1) and CCMP631 (Pt6). **Figure S5.** Depth of base calls at each nucleotide position of the 5’ end of *VDL1* gene based on the published RNA sequencing data (Mccarthy et al., 2017) of *P. tricornutum* strain CCMP2561 (Pt1). **Figure S6.** Predicted structure of VDL1 by AlphaFold. **Figure S7.** The analysis of allele-specific expression (ASE) of *VDL1* based on the published RNA sequencing data (Mccarthy et al., 2017) of *P. tricornutum* strain CCMP2561 (Pt1). **Figure S8.** Sequence analysis of *VDL1* promoters from *P. tricornutum* strains Pt1 and Pt6 by PlantCare. **Figure S9.** Schematic representations of the expression constructs used in this study. **A.** Overexpression vector of *PtVDL1*. **B.** *eGFP* fusion vector of *PtVDL1*.

**Additional file 2: Table S1.** Average depth of base calls at each nucleotide position of the 5’ end (-203 ~ 100 bp) of *P. tricornutum VDL1* gene based on the published RNA sequencing data (Mccarthy et al., 2017). **Table S2.** RPKM values of *VDL1* alleles at different culture conditions based on the reanalysis of RNA sequencing data from Mccarthy et al. (2017). **Table S3.** Identity analysis of VDL1 proteins from different species. **Table S4.** Signal peptide prediction results. **Table S5.** Percentage of pigment contents (data from Fig. 6b) in wild-types Pt1 and Pt6, and in eight *VDL1* overexpression (OE) strains. **Table S6.** Information on *VDL1* polymorphic sites, and software running parameters and version. **Table S7.** Promoter elements of *VDL1* gene. **Table S8.** Primers used in the present study.

## Data Availability

All data generated or analyzed during this study are included in this published article and its supplementary information files.
