# Supplementary material for: A key gene, violaxanthin de-epoxidase-like 1, enhances fucoxanthin accumulation in Phaeodactylum tricornutum
Source: Biotechnol Biofuels Bioprod. 2024 Apr 2;17:49. doi: 10.1186/s13068-024-02496-3 (PMC10986045; doi:10.1186/s13068-024-02496-3)
Supplement: Supplementary file 1 — Additional file 1: Figure S1. Fucoxanthin content in different accessions of P. tricornutum. Figure S2. Phylogenetic association of VDL1 gene in different accessions of P. tricornutum based on polymorphic sites (including SNP and INDELS) using a maximum likelihood approach. Figure S3. Single nucleotide variants (SNVs) of the VDL1 gene in P. tricornutum strains CCMP2561 (Pt1) and CCMP631 (Pt6). Figure S4. Sequence alignment of the amino acids of VDL1 in P. tricornutum strains CCMP2561 (Pt1) and CCMP631 (Pt6). Figure S5. Depth of base calls at each nucleotide position of the 5’ end of VDL1 gene based on the published RNA sequencing data (Mccarthy et al., 2017) of P. tricornutum strain CCMP2561 (Pt1). Figure S6. Predicted structure of VDL1 by AlphaFold. Figure S7. The analysis of allele-specific expression (ASE) of VDL1 based on the published RNA sequencing data (Mccarthy et al., 2017) of P. tricornutum strain CCMP2561 (Pt1). Figure S8. Sequence analysis of VDL1 promoters from P. tricornutum strains Pt1 and Pt6 by PlantCare. Figure S9. Schematic representations of the expression constructs used in this study. A. Overexpression vector of PtVDL1. B. eGFP fusion vector of PtVDL1. [file 13068_2024_2496_MOESM1_ESM.pdf]

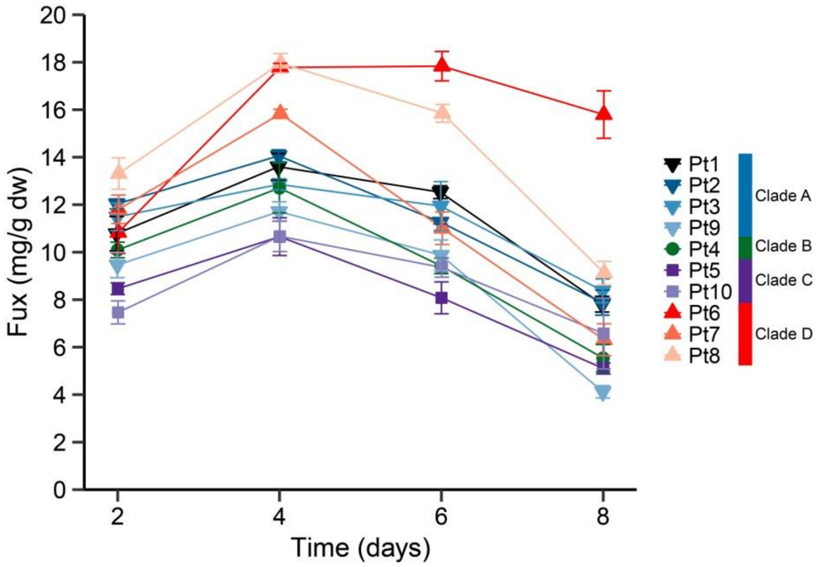

Fig. S1 Fucoxanthin content in different accessions of *P. tricornutum*.

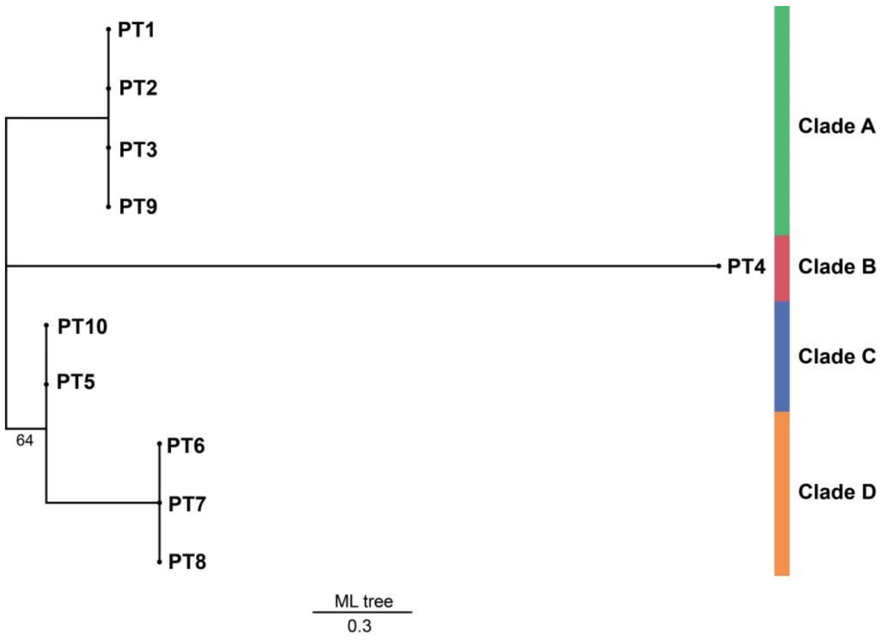

Fig. S2 Phylogenetic association of *VDL1* gene in different accessions of *P. tricornutum* based on polymorphic sites (including SNP and INDELS) using a maximum likelihood approach. The four genetic clades (Genotype A–D) of *P. tricornutum* have been described in Rastogi et al. (2020).

```
                          10        20        30        40        50        60        70        80        90
                  ....|....|....|....|....|....|....|....|....|....|....|....|....|....|....|....|....|....|
Phatr3_J36048     CGGAATGTGCTTCGCTGTCATCGTTATAGAGCTGTGCTTCTTGACTTTTGGAAACTTCATACTGTCGGAAGAGTTCTCGTTCCGTGACCC
Pt1VDL1-allele 1  .......................................................................................T..
Pt1VDL1-allele 2  ...G......................................................G...............................
Pt6VDL1-allele 1  ..........................................................................................
Pt6VDL1-allele 2  ..........................................................................................

                         100       110       120       130       140       150       160       170       180
                  ....|....|....|....|....|....|....|....|....|....|....|....|....|....|....|....|....|....|
Phatr3_J36048     ATTCCAAGTCTGTTCCGCTAGAGGATTGCTGCGGGTCTTCCGTAGGATTGGCATCCCCCAGGACTTCTTCGCGTTGGAGGGCCGCCTCGA
Pt1VDL1-allele 1  ..........................................................................................
Pt1VDL1-allele 2  ..........................................................................................
Pt6VDL1-allele 1  ..........................................................................................
Pt6VDL1-allele 2  ..........................................................................................

                         190       200       210       220       230       240       250       260       270
                  ....|....|....|....|....|....|....|....|....|....|....|....|....|....|....|....|....|....|
Phatr3_J36048     CCAAATCCGTCTTGGCGTGATACACCGACAGGTACGGCACCGTCCAGTGCTCGTTGGTTTCCTCGCCGTAATGTCCGTGACAGCAGCGTC
Pt1VDL1-allele 1  ..........................................................................................
Pt1VDL1-allele 2  .........................................................................C................
Pt6VDL1-allele 1  ..........................................................................................
Pt6VDL1-allele 2  ..........................................................................................

                         280       290       300       310       320       330       340       350       360
                  ....|....|....|....|....|....|....|....|....|....|....|....|....|....|....|....|....|....|
Phatr3_J36048     GGTGCCACAAGGTCCCTTCGTGCGGTTCGAACCAGAGCCAGTCGGCCGGAATGCGCGCTTGGCGGGCACGGGAACAGCGACAGACGGCGC
Pt1VDL1-allele 1  ..........................................................................................
Pt1VDL1-allele 2  ..........................................................................................
Pt6VDL1-allele 1  ..........................................................................................
Pt6VDL1-allele 2  ......................................T...................................................

                         370       380       390       400       410       420       430       440       450
                  ....|....|....|....|....|....|....|....|....|....|....|....|....|....|....|....|....|....|
Phatr3_J36048     AACAAAACGGAGCCAACAAGGCAAATTCCTCGTCACTGGGCAAATCTAGGAGTGCCGCTCCTTCCTCCGCATCCGTATTTACCAGTCGTT
Pt1VDL1-allele 1  ..........................................................................................
Pt1VDL1-allele 2  ........................................................................................C.
Pt6VDL1-allele 1  ........................................................................................C.
Pt6VDL1-allele 2  ..........................................................................................

                         460       470       480       490       500       510       520       530       540
                  ....|....|....|....|....|....|....|....|....|....|....|....|....|....|....|....|....|....|
Phatr3_J36048     CGTCGCGGGATAAGCGAGCGAGTACACTACGAGCAGTCCGACGGCCTTGGCGGACTTCGTTGCTTTCCGTCCGGTCATCGTCATCCGGTG
Pt1VDL1-allele 1  ..........................................................................C...............
Pt1VDL1-allele 2  ..........................................................................................
Pt6VDL1-allele 1  ..........................................................................................
Pt6VDL1-allele 2  ..........................................................................................

                         550       560       570       580       590       600       610       620       630
                  ....|....|....|....|....|....|....|....|....|....|....|....|....|....|....|....|....|....|
Phatr3_J36048     GGGAATCGTACGGGTCGGGGTCGACGCGTGGGTCCGGGACACGCCGGACCACACGCGCTCGTTTCGCTCGACGTTGTCTCATTCCTTTTC
Pt1VDL1-allele 1  ......................................................................T...................
Pt1VDL1-allele 2  ..........................................................................................
Pt6VDL1-allele 1  ..........................................................................................
Pt6VDL1-allele 2  ......................................................................T...................

                         640       650       660       670       680       690       700       710       720
                  ....|....|....|....|....|....|....|....|....|....|....|....|....|....|....|....|....|....|
Phatr3_J36048     GAAACAAGCACAAGTACTACACTCACATGCACTGTTTCTACCGCCTGGGAGGCAGTCGCCACGAATAAACGCTGAACAGGGCGACTATAG
Pt1VDL1-allele 1  ........................................T.A...............G...........A...................
Pt1VDL1-allele 2  ..........................................................................................
Pt6VDL1-allele 1  ........................................T.A...............G...............................
Pt6VDL1-allele 2  ..........................................................................................

                         730       740       750       760       770       780       790       800       810
                  ....|....|....|....|....|....|....|....|....|....|....|....|....|....|....|....|....|....|
Phatr3_J36048     TGTGTTCTACCTTTGCTTTGGATGGTATAGTTCCCGCACGTTGCATGGTCCACGATGCCAAACTCACTCTTGACA---------------
Pt1VDL1-allele 1  .............................................C.............................ATAACTGTAAAGTCA
Pt1VDL1-allele 2  ...........................................................................---------------
Pt6VDL1-allele 1  .............................................C........G....................ATAACTGTAAAGTCA
Pt6VDL1-allele 2  ............................G..............................................---------------

                         820       830       840       850       860       870       880       890       900
                  ....|....|....|....|....|....|....|....|....|....|....|....|....|....|....|....|....|....|
Phatr3_J36048     ------TACTCTTACAGTTAGTGTGACATTTGAGGCGTTTTCTTGGTAACAATTACCTATGCCCTGCGAATTCAAAGTCCGGGTTTCAAA
Pt1VDL1-allele 1  GGAGGG....................................................................................
Pt1VDL1-allele 2  ------....................................................................................
Pt6VDL1-allele 1  GGAGGG....................................................................................
Pt6VDL1-allele 2  ------....................................................................................
```

```
                         910       920       930       940       950       960       970       980       990
                  ....|....|....|....|....|....|....|....|....|....|....|....|....|....|....|....|....|....|
Phatr3_J36048     TGGTCACAATCGGTTCAAATTACAGCTTGGACGTGTTGACTGTGATCACAGATACGTTAACGTACCGTTAAAACTATGCCTGGAGCCGAG
Pt1VDL1-allele 1  ............................................G.............................................
Pt1VDL1-allele 2  .A........................................................................................
Pt6VDL1-allele 1  C..G....T.................................................................................
Pt6VDL1-allele 2  .A........................................................................................

                        1000      1010      1020      1030      1040      1050      1060      1070      1080
                  ....|....|....|....|....|....|....|....|....|....|....|....|....|....|....|....|....|....|
Phatr3_J36048     CCCTATGTGACCACGGTCTATAGATGTCGGGTTCAACTCGGCGTCTGTCTCCCGGACTGTGACGGGGATAAACCGAAAATCCTCCGTCGT
Pt1VDL1-allele 1  ..........................................................................................
Pt1VDL1-allele 2  ...........................A..................----.................G......................
Pt6VDL1-allele 1  ...........................A..................----.................G......................
Pt6VDL1-allele 2  ...........................A..................----.................G......................

                        1090      1100      1110      1120      1130      1140      1150      1160      1170
                  ....|....|....|....|....|....|....|....|....|....|....|....|....|....|....|....|....|....|
Phatr3_J36048     GCCATCACCATATCGCATCGCGTCAATCCAATCCTAACACTGGCGTTTCTACTAGGAGGGACCCCGTTACACAACGTCAAAGCGTATCCA
Pt1VDL1-allele 1  ..........................................................................................
Pt1VDL1-allele 2  .......................................................A............G.....................
Pt6VDL1-allele 1  ....................................................................G.....................
Pt6VDL1-allele 2  .......................................................A............G.....................

                        1180      1190      1200      1210      1220      1230      1240      1250      1260
                  ....|....|....|....|....|....|....|....|....|....|....|....|....|....|....|....|....|....|
Phatr3_J36048     CGGGGGAAAATGCGATTCGCTTGGGTGGTAGCAGCCGGAGTCGTCTTGACGACGACGACACAAGCCTTGGTGCCTTTAGATTGCACGGGT
Pt1VDL1-allele 1  ..........................................................................................
Pt1VDL1-allele 2  ........................................................................T.................
Pt6VDL1-allele 1  ..........................................................................................
Pt6VDL1-allele 2  ..........................................................................................

                        1270      1280      1290      1300      1310      1320      1330      1340      1350
                  ....|....|....|....|....|....|....|....|....|....|....|....|....|....|....|....|....|....|
Phatr3_J36048     ATGGGTGAAACAAGAACGAGCGGCATCCGTCCGATCCGGGGTCTGGAAAGCAACATGGCCCGGTACGCTACTGTACGCCACGGCACCGAC
Pt1VDL1-allele 1  .......................................A..................................................
Pt1VDL1-allele 2  ..........................................................................................
Pt6VDL1-allele 1  ..................C.......................................................................
Pt6VDL1-allele 2  ..........................................................................................

                        1360      1370      1380      1390      1400      1410      1420      1430      1440
                  ....|....|....|....|....|....|....|....|....|....|....|....|....|....|....|....|....|....|
Phatr3_J36048     CAGACGAACCACGGCATAACTTCCTCTTCTGAAAGACAGTGGCCTTTTCCACGCGGGGGTTCTTCTCCCAGAGCGGTAGCGAGGTCCGTG
Pt1VDL1-allele 1  ..........................................................................................
Pt1VDL1-allele 2  ................GTG.......................................................................
Pt6VDL1-allele 1  ..........................................................................................
Pt6VDL1-allele 2  ................GTG.......................................................................

                        1450      1460      1470      1480      1490      1500      1510      1520      1530
                  ....|....|....|....|....|....|....|....|....|....|....|....|....|....|....|....|....|....|
Phatr3_J36048     GCGACGTTCGGACTCGGTTTCTCCATCGCACTGGCGAGTGTTTTCGGAGTCGCGGCGGCTCCCGTCGGGGCCGACACGACTCCCGCCGTC
Pt1VDL1-allele 1  ..............................................................G.....C.....................
Pt1VDL1-allele 2  ..........................................................................................
Pt6VDL1-allele 1  ..........................T...............................................................
Pt6VDL1-allele 2  ..........................................................................................

                        1540      1550      1560      1570      1580      1590      1600      1610      1620
                  ....|....|....|....|....|....|....|....|....|....|....|....|....|....|....|....|....|....|
Phatr3_J36048     AAGTACGACGGATTCGCAGAATACGCTCAAGATAACCAAATGGAGCAATCTGACGTCGGATGCTTTATCAATAAATGTGGTGATCAAACC
Pt1VDL1-allele 1  ...................................T......................................................
Pt1VDL1-allele 2  ..................................................C.......................................
Pt6VDL1-allele 1  ...................................T..............C.......................................
Pt6VDL1-allele 2  ..................................................C.......................................

                        1630      1640      1650      1660      1670      1680      1690      1700      1710
                  ....|....|....|....|....|....|....|....|....|....|....|....|....|....|....|....|....|....|
Phatr3_J36048     AAGGCTCTCTTTAGCAATCCGCGTGGAATCAAAGGTGTCTCCTGTTTGGGACGCTGCAAGGGCGAGCAATCGTGTGCGACGCGGTGTTTT
Pt1VDL1-allele 1  .....A....................................................................................
Pt1VDL1-allele 2  ..........................................................................................
Pt6VDL1-allele 1  .....A....................................................................................
Pt6VDL1-allele 2  ..........................................................................................

                        1720      1730      1740      1750      1760      1770      1780      1790      1800
                  ....|....|....|....|....|....|....|....|....|....|....|....|....|....|....|....|....|....|
Phatr3_J36048     GCCGAATTCGGAAGCGAGAGCTTAAACGCATGGTTGTCCTGTACTATCGAGGAAAATGAGTGCGTCAAGGTCCCGAAAAATGTCGACAAC
Pt1VDL1-allele 1  ........................................................C.................................
Pt1VDL1-allele 2  ............................................G.............................................
Pt6VDL1-allele 1  ............................................G.............................................
Pt6VDL1-allele 2  ............................................G.............................................
```

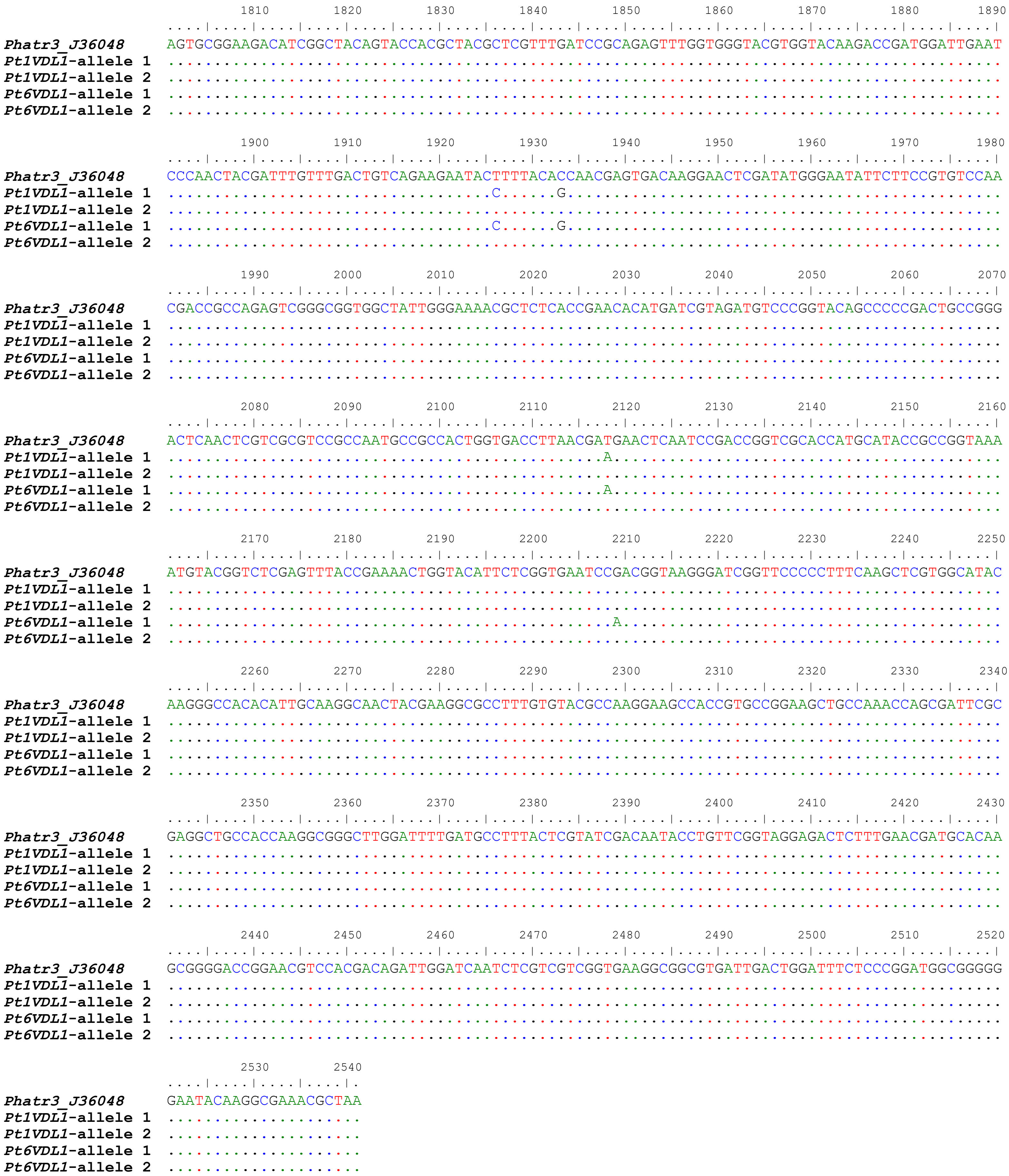

Fig. S3 Single nucleotide variants (SNVs) of the *VDL1* gene in *P. tricornutum* strains CCMP2561 (Pt1) and CCMP631 (Pt6). Allele 1 and allele 2 represent the *VDL1* alleles 1 and 2, respectively. 4-bp deletion is indicated in the red box and translation start site in the blue box. *Phatr3_J36048* is the sequence obtained from Ensembl.

```
                            10        20        30        40        50        60        70
                    ....|....|....|....|....|....|....|....|....|....|....|....|....|....|
Phatr3_J36048       MPGAEPYVTTVYRCRVQLGVCLPDCDGDKPKILRRAITISHRVNPILTLAFLLGGTPLHNVKAYPRGKMR
Pt1VDL1-allele 1    ......................................................................
Pt1VDL1-allele 2    --------------------------------------------------------------------..
Pt6VDL1-allele 1    --------------------------------------------------------------------..
Pt6VDL1-allele 2    --------------------------------------------------------------------..

                            80        90       100       110       120       130       140
                    ....|....|....|....|....|....|....|....|....|....|....|....|....|....|
Phatr3_J36048       FAWVVAAGVVLTTTTQALVPLDCTGMGETRTSGIRPIRGLESNMARYATVRHGTDQTNHGITSSSERQWP
Pt1VDL1-allele 1    ......................................S...............................
Pt1VDL1-allele 2    ...................S........................................SA........
Pt6VDL1-allele 1    ...............................R......................................
Pt6VDL1-allele 2    ............................................................SA........

                           150       160       170       180       190       200       210
                    ....|....|....|....|....|....|....|....|....|....|....|....|....|....|
Phatr3_J36048       FPRGGSSPRAVARSVATFGLGFSIALASVFGVAAAPVGADTTPAVKYDGFAEYAQDNQMEQSDVGCFINK
Pt1VDL1-allele 1    ......................................................................
Pt1VDL1-allele 2    ......................................................................
Pt6VDL1-allele 1    ......................................................................
Pt6VDL1-allele 2    ......................................................................

                           220       230       240       250       260       270       280
                    ....|....|....|....|....|....|....|....|....|....|....|....|....|....|
Phatr3_J36048       CGDQTKALFSNPRGIKGVSCLGRCKGEQSCATRCFAEFGSESLNAWLSCTIEENECVKVPKNVDNSAEDI
Pt1VDL1-allele 1    ......................................................................
Pt1VDL1-allele 2    ......................................................................
Pt6VDL1-allele 1    ......................................................................
Pt6VDL1-allele 2    ......................................................................

                           290       300       310       320       330       340       350
                    ....|....|....|....|....|....|....|....|....|....|....|....|....|....|
Phatr3_J36048       GYSTTLRSFDPQSLVGTWYKTDGLNPNYDLFDCQKNTFTPTSDKELDMGIFFRVQRPPESGGGYWENALT
Pt1VDL1-allele 1    .......................................A..............................
Pt1VDL1-allele 2    ......................................................................
Pt6VDL1-allele 1    .......................................A..............................
Pt6VDL1-allele 2    ......................................................................

                           360       370       380       390       400       410       420
                    ....|....|....|....|....|....|....|....|....|....|....|....|....|....|
Phatr3_J36048       EHMIVDVPVQPPTAGTQLVASANAATGDLNDELNPTGRTMHTAGKMYGLEFTENWYILGESDGKGSVPPF
Pt1VDL1-allele 1    ..............................E.......................................
Pt1VDL1-allele 2    ......................................................................
Pt6VDL1-allele 1    ..............................E..............................N........
Pt6VDL1-allele 2    ......................................................................

                           430       440       450       460       470       480       490
                    ....|....|....|....|....|....|....|....|....|....|....|....|....|....|
Phatr3_J36048       KLVAYKGHTLQGNYEGAFVYAKEATVPEAAKPAIREAATKAGLDFDAFTRIDNTCSVGDSLNDAQAGTGT
Pt1VDL1-allele 1    ......................................................................
Pt1VDL1-allele 2    ......................................................................
Pt6VDL1-allele 1    ......................................................................
Pt6VDL1-allele 2    ......................................................................

                           500       510       520
                    ....|....|....|....|....|....|.
Phatr3_J36048       STTDWINLVVGEGGVIDWISPGWRGEYKAKR
Pt1VDL1-allele 1    ...............................
Pt1VDL1-allele 2    ...............................
Pt6VDL1-allele 1    ...............................
Pt6VDL1-allele 2    ...............................
```

Fig. S4 Sequence alignment of the amino acids of VDL1 in *P. tricornutum* strains CCMP2561 (Pt1) and CCMP631 (Pt6). Allele 1 and allele 2 represent the *VDL1* alleles 1 and 2 encoding amino acid sequences respectively, and Phatr3_J36048 is the sequence obtained from Ensembl.

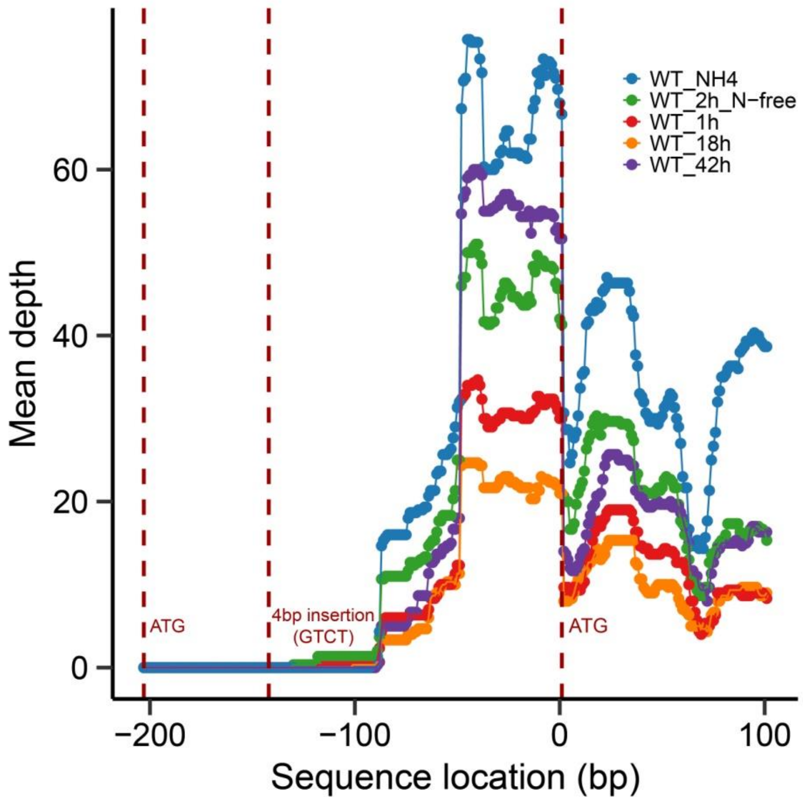

Fig. S5 Depth of base calls at each nucleotide position of the 5' end of *VDL1* gene based on the published RNA sequencing data (Mccarthy et al., 2017) of *P. tricornutum* strain CCMP2561 (Pt1). WT_NH4 stands for mid-exponential phase cells with 880 μM NH_4^+; WT_2h N-free stands for 2 h of incubation in N-free media; and WT_1h, 18h, 42h stand for 1~42 h after the transfer of cultures into 300 μM NO_3^- media.

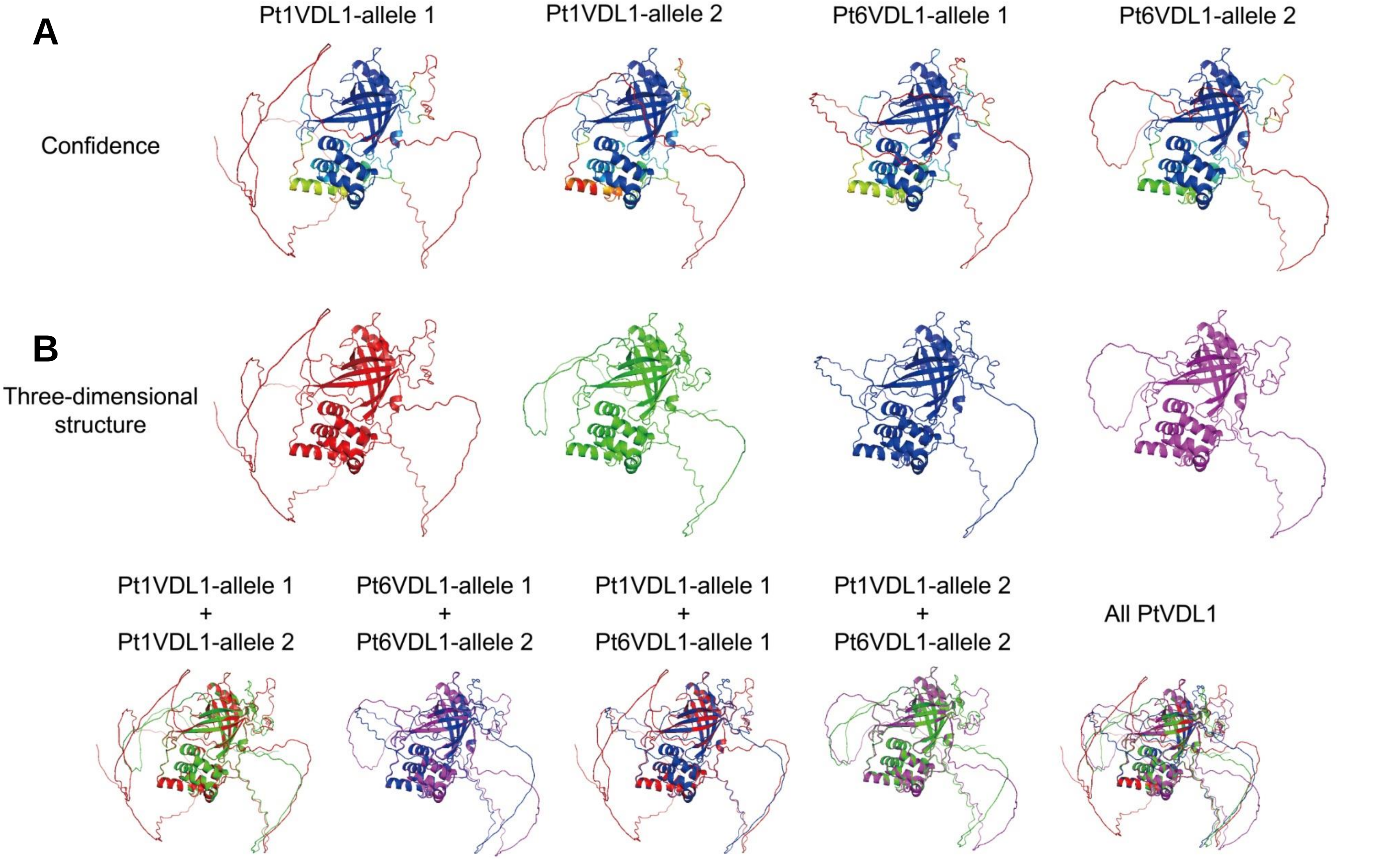

Fig. S6 Predicted structure of VDL1 by AlphaFold. **A.** Predicted structure of VDL1s coloured according to the per-residue confidence score (predicted local distance difference test, pLDDT), very low: red (pLDDT < 50), low: yellow (70 > pLDDT > 50), confident: cyan (90 > pLDDT > 70), very high: blue (pLDDT > 90). **B.** Comparison of predicted structure of VDL1s. VDL1-allele 1 and VDL1-allele 2: VDL1 proteins encoded by alleles 1 and 2. Pt1, *P. tricornutum* strain CCMP2561; Pt6, *P. tricornutum* strain CCMP631.

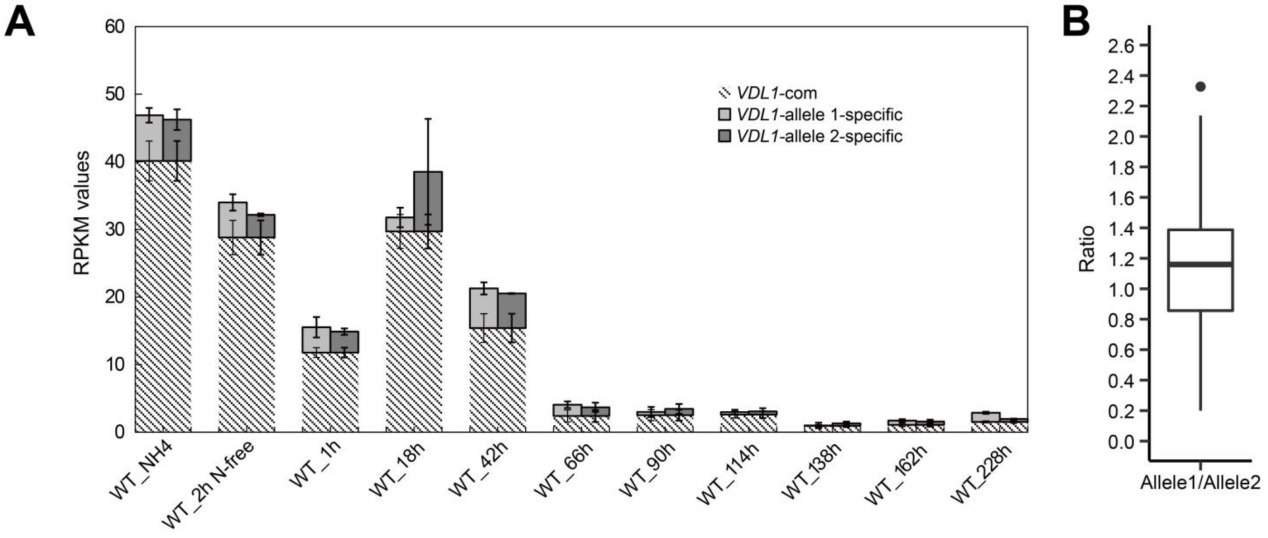

Fig. S7 The analysis of allele-specific expression (ASE) of *VDL1* based on the published RNA sequencing data (Mccarthy et al., 2017) of *P. tricornutum* strain CCMP2561 (Pt1). **A.** RPKM values of two alleles in response to nitrogen stress and culture time. **B.** Average ratio of RPKM values of allele 1 versus those of allele 2. *VDL1*-com refers to the reads mapped to the regions with no difference between *VDL1* alleles; *VDL1*-allele 1-specific and *VDL1*-allele 2-specific refer to the reads specifically mapped to the two *VDL1* alleles 1 and 2, respectively. WT_NH4 stands for mid-exponential phase cells with 880 μM NH_4^+; WT_2h N-free stands for 2 h of incubation in N-free media; and WT_1h, 18h, 42h, 66h, 90h, 114h, 138h, 162h, 228h stand for 1~228 h after the transfer of cultures into 300 μM NO_3^- media.

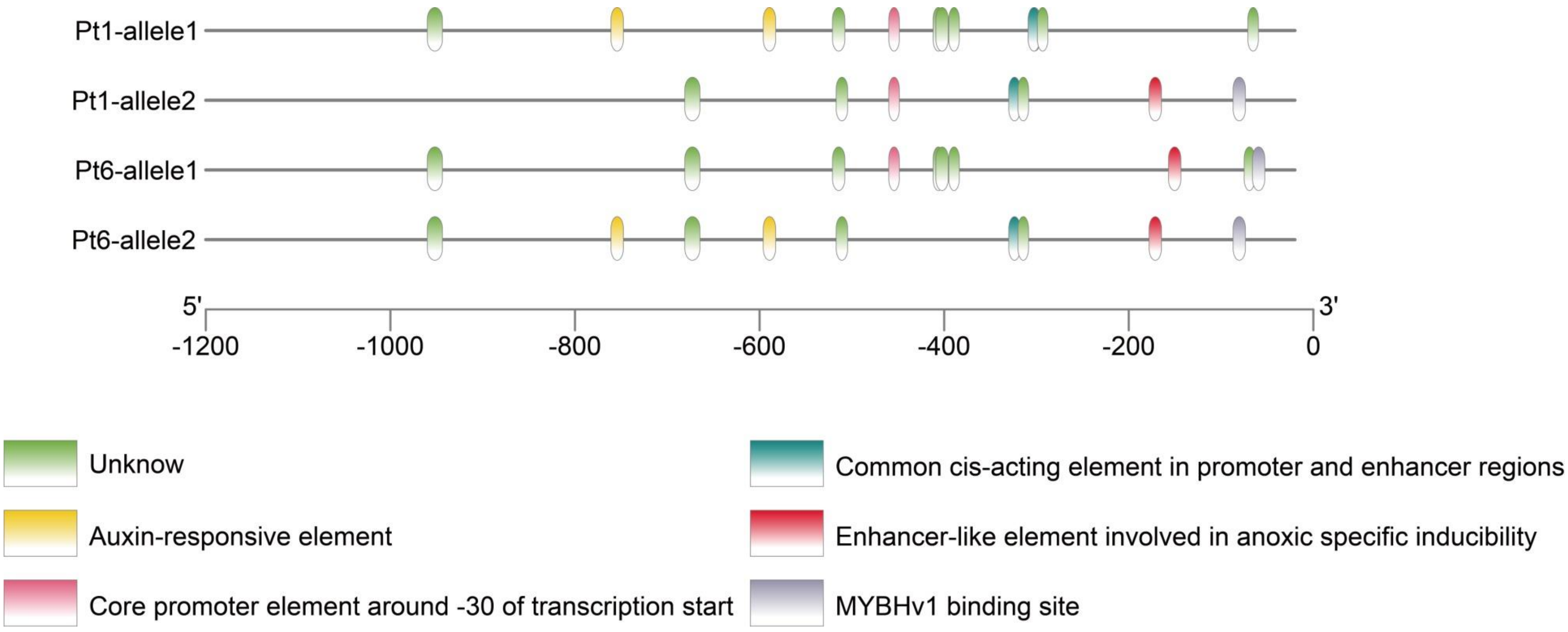

Fig. S8 Sequence analysis of *VDL1* promoters from *P. tricornutum* strains Pt1 and Pt6 by PlantCare. Different potentially functional elements between the two strains were shown. Pt1-allele1 and Pt1-allele2, Pt6-allele1 and Pt6-allele2: promoters of *VDL1* alleles from Pt1 and Pt6, respectively.

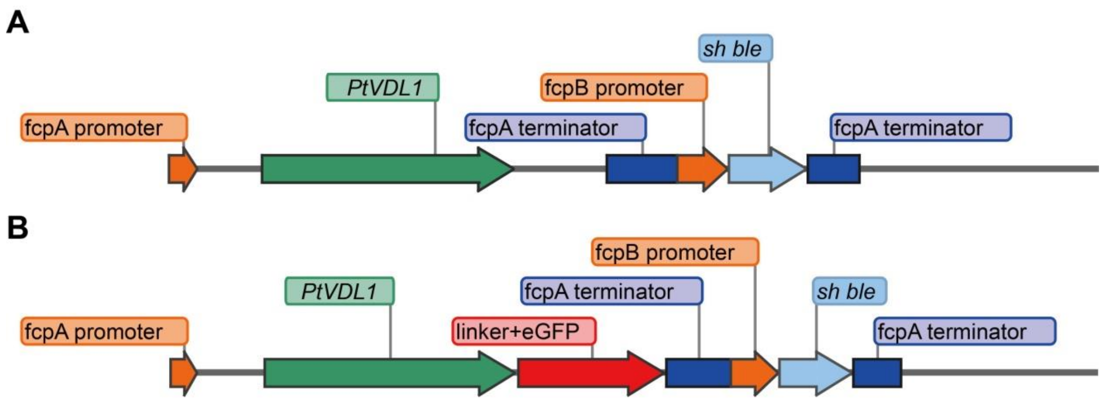

Fig. S9 Schematic representations of the expression constructs used in this study. **A.** Overexpression vector of *PtVDL1*. **B.** *eGFP* fusion vector of *PtVDL1*.
